# Sex Differences in the Blood Oxygen Level-Dependent Signal to Placebo Analgesia and Nocebo Hyperalgesia in Experimental Pain: A Functional MRI Study

**DOI:** 10.3389/fnbeh.2021.657517

**Published:** 2021-08-23

**Authors:** Yu Shi, Hongrui Zhan, Yanyan Zeng, Shimin Huang, Guiyuan Cai, Jianming Yang, Wen Wu

**Affiliations:** ^1^Department of Rehabilitation, Zhujiang Hospital, Southern Medical University, Guangzhou, China; ^2^Rehabilitation Medical School, Southern Medical University, Guangzhou, China; ^3^Department of Rehabilitation, The Fifth Affiliated Hospital of Sun Yat-sen University, Zhuhai, China; ^4^Department of Radiology, Zhujiang Hospital, Southern Medical University, Guangzhou, China

**Keywords:** placebo, nocebo, sex differences, functional connectivity, reward system, anxiety

## Abstract

**Objective:**

Placebo as well as nocebo responses are widely found in scientific research and clinical practice. Growing evidence suggests sex differences in placebo as well as nocebo responses. However, data concerning this question are still insufficient. This study examined whether the BOLD signals of two responses, as measured with functional MRI (fMRI), differ by sex under conditions of equivalent experimental pain perception.

**Method:**

Thirty-one healthy volunteers (14 female) underwent two fMRI scans, once during a placebo intervention and once during a nocebo intervention, pseudorandomly ordered, in an acute lower back pain (ALBP) model. We collected visual analog scale (VAS) data after each scanning. fMRI data from different sex groups were subjected to functional connectivity (FC) analysis and behavioral correlation analysis (BCA).

**Results:**

The results showed statistical differences in VAS scores between male and female participants, in both placebo and nocebo responses. Both groups also showed reduced FC in the pain-associated network of the placebo response and elevated FC in the pain-related network of the nocebo response. However, in the placebo condition, male participants displayed increased FC in the ventromedial prefrontal cortex, parahippocampal gyrus (PHP), and posterior cingulate cortex (PCC), while female participants showed increased FC in the dorsolateral prefrontal cortex, hippocampal gyrus (HP), and insular cortex (IC). In the nocebo condition, male participants showed decreased FC in the PCC and HP, while female participants displayed decreased FC in the mid-cingulate cortex, thalamus (THS), and HP. The BCA results of the two groups were also different.

**Conclusion:**

We found that the endogenous opioid system and reward circuit play a key role in sex differences of placebo response and that anxiety and its secondary reactions may cause the sex differences of nocebo response.

## Introduction

Placebo and nocebo responses have been given more and more attention in clinical practice and scientific research, especially in the research area of pain (Jensen et al., 2012; Testa and Rossettini, 2016). The placebo effect is the reduction of pain based on the belief or expectation that pain will be reduced by some drug or treatment, whereas the nocebo effect entails the worsening of pain or other symptoms when treatment is not expected to work well (Benedetti, 1996).

The performance of placebo analgesia as well as nocebo hyperalgesia varies greatly among individuals, with sex considered as may be an important factor behind the differences (Vambheim and Flaten, 2017). Some studies have reported sex differences in the two responses (Aslaksen et al., 2011; Swider and Babel, 2013), while other researchers believe that no such differences exist (Olofsen et al., 2005). Such contradictory conclusions suggest that we need further research on the sex effects on the two responses. As research has confirmed that sex effects play an important role in pain (Garcia et al., 2007), we hypothesize that sex effects also have a vital role in placebo as well as nocebo responses.

With the development of brain functional imaging technology, such method has become a key measure for studying neuroscience (Amanzio et al., 2013; Freeman et al., 2015). Theysohn et al. (2015) reported that women as well as men exhibited similar placebo response at the subjective pain assessment level but different activation of the insular cortex (IC) and dorsolateral prefrontal cortex (DLPFC). Schmid et al. (2015) reported that the regions of the thalamus (THS), IC, amygdala (AMYG), and mid-cingulate cortex (MCC) have a critical role in the nocebo hyperalgesia brain network.

To date, in the research area of placebo and nocebo responses, only few researchers focused on the sex differences of placebo response (Theysohn et al., 2015; Benedetti, 2016). No studies have explored the sex differences of nocebo. There was also no study that explored both placebo along with nocebo influences in the same group of subjects. Therefore, further research is needed.

In addition, the role of hormones associated with the menstrual cycle, such as estradiol and progesterone, in the placebo effect should also be noted (McEwen and Milner, 2017). Previous studies have shown that estradiol affects multiple neurotransmitter release systems, such as dopamine, serotonin, and gamma-aminobutyric acid (GABA), and modulates the executive function of the brain throughout the menstrual cycle (Poromaa et al., 2003). The above neurotransmitters are closely related to the placebo/nocebo effects. The release and regulation of dopamine and GABA can significantly change the intensity and efficiency of the placebo effect. Progesterone during the cycle can also be metabolized into the neurosteroid to enhance GABA receptors, thus acting as an anti-anxiety and sedative, which may enhance the placebo effect (Melcangi et al., 2011). At the same time, progesterone also affects the release of transmitters in the hypothalamic–pituitary–adrenal (HPA) axis, which may have an impact on emotional memory and pain memory during pain (Andreano et al., 2008; Andreano and Cahill, 2010). Therefore, hormone levels at different phases of the menstrual cycle have a significant influence on the placebo/nocebo effects. In order to reduce the effect of menstrual hormones on the placebo/nocebo effects, the period of low hormone release should be selected as the observation point, and mid and late follicular phase (the period after the menstrual phase) is a better choice. During this period of time, the release level of hormones is lower, and the emotional impact of premenstrual syndrome can also be avoided.

Given that the rostral anterior cingulate cortex (rACC) is extensively linked to the IC and the cerebral sensory cortex and functions in the receiving and coming out sensory information from pain signaling pathways (Craig et al., 2000), the rACC is also a key component of the reward pathway (Price, 2000). Hence, the choice of the rACC as a region of interest (ROI) is an essential means of studying the pivotal regions of the network of the brain.

Herein, we used an established model of acute lower back pain (ALBP) (16) to assess sex effects in placebo as well as nocebo responses. The intervention expectations of the participants of the effectiveness of two patches were manipulated by labeling one an “analgesic patch” (placebo intervention) and the other an “algetic patch” (nocebo intervention). We then examined the self-reported pain scores of the participants and changes in the blood oxygen level-dependent (BOLD) signals before and after the different “interventions.” This is the first time that sex differences in two effects have been compared directly, under conditions of equivalent experimental pain perception. With this research, we expect to increase our understanding of the mechanisms of placebo as well as nocebo responses.

## Materials and Methods

### Participants

Herein, the participants were recruited through advertisements, and the participants were all right-handed. Inclusion criteria were as follows: (i) candidate had not participated in prior psychological experiments; (ii) candidate body mass index should be in normal weight (18.5–23.9); (iii) candidate had not had psychiatric or medical conditions, including depression and mania in the previous 4 weeks; (iv) candidate did not have pain, including dysmenorrhea, and should not have been on medication, including antipyretics and sleeping pills in the previous 4 weeks; (v) female candidate should be in the mid and late follicular phase; and (vi) candidates scored <50 on the self-rating anxiety scales (SAS) and the self-rating depression scales (SDS) (a score of <50 represents “candidate mentally normal”). Candidates were excluded if they had (i) organic brain disease, (ii) history of craniocerebral injury, (iii) drug dependence, (iv) severe neurological disorder, (v) metal component in body, (vi) claustrophobia, or (vii) taken pain killers in the previous 4 weeks. The Ethics Committee of Zhujiang Hospital affiliated to Southern Medical University approved all the experiments as well as the protocols (World Medical Association, 2013). After the experiment is completed, we will introduce the real purpose of the study to the participants. All participants were provided with the option of withdrawing their data from the study in the case of any issues relating to the methodological requirement for deception in the experimental paradigm. No participant reported any issue; therefore, all permitted the use of their data.

### Experimental Procedures

Herein, two patches were designed for conveying the psychological suggestions: we labeled one as “analgesic patch” (positive expectancy), while the second one was labeled as “algetic patch” (negative expectancy).

**FIGURE 1:**
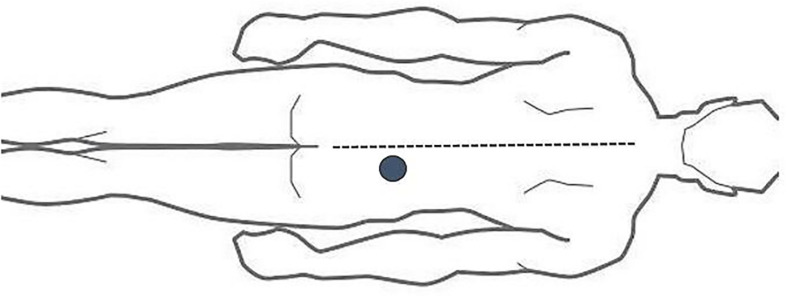
ALBP model location.

The ALBP model employed was according to a previous study (Shi et al., 2015). Based on the model, we established an injection point 2 cm lateral to the neural spine of the fourth lumbar vertebra. After that, 10 ml of 5% sterile hypertonic saline was filled into a 24-gauge indwelling needle, which was then attached into a computer-controlled power injector (Spectris Solaris EP; Medrad, Inc., Warrendale, PA, United States) through a long connecting tube, followed by its vertical insertion into the abovementioned area at 1.5 cm depth. After 1 min, the hypertonic saline was administered intramuscularly into the ALBP participant. The injection consisted of bolus administration (0.1 ml within 5 s) and successive continuous injections (0.15 ml/min) to induce persistent ALBP (Figure 1).

#### Training Session

Here, we familiarized the study subjects with the ALBP and the visual analog scales (VASs), which they could use to grade their pain. Assessment of the intensity of the pain was on the basis of a 10-cm VAS anchored with “no pain” (0) and “worst pain imaginable” (10). The unpleasantness of the pain (i.e., distressing as well as horrible) was evaluated by a 10-cm in-house mood scale anchored with “infinitely small” (0) and “excruciating” (10). Besides, we recorded any experienced discomfort by the participants to avoid adverse reactions.

#### Behavioral Conditioning Session

All the study subjects were informed that the aim of the study involved exploring the analgesic impacts of the analgesic patch as well as the algetic impacts of the algetic patch. The participants were informed that one of the two patches (the algetic patch or the analgesic patch) would be applied to their right foot when they have an ALBP. After that, the participant should expect to experience change in pain based on the applied patch; the order of patch application is random.

After the application of the patch, the experiment was manipulated. During this conditioning paradigm, the subjects were informed that they would experience a pain change based on if they had an analgesic patch or an algetic patch. Within that process, we instructed the participants to focus on the screen captions. If the participants had an analgesic patch, the captions projected on the screen included “Please experience the effect of the analgesic patch,” and when participants had an algetic patch, the caption projected included “Please experience the effect of the algetic patch.” After the application of each stimulus, we projected the VAS on the screen, and the participants reported their pain scores. In reality, we reduced the speed of hypertonic saline where the participant had an analgesic patch and increased the speed of hypertonic saline when the participant had an algetic patch. Only the study subjects who could differentiate between the pre- and post-intervention of the analgesic impacts of the analgesic patch and the algetic impacts of the algetic patch were permitted to continue with the study.

#### Scan Session

The study subjects were informed that the events of the scan session would be similar to those of the previous session. In fact, we designed the scan session to assess the nocebo and placebo responses triggered by the expectancy manipulation in the behavioral conditioning session. Therefore, the scan session process was similar to that of the behavioral conditioning session. That is to say, functional MRI (fMRI) scanning is completed simultaneously on the basis of the original behavioral conditioning session.

First, the brain anatomical scans were collected before the fMRI scans. At the beginning, the participants were subjected to a normal (baseline) fMRI scan for 6 min. We induced a preliminary ALBP model in the right lower back muscle of each subject, as above. In the first 6 min of the ALBP condition, an fMRI scan was conducted to assess the pain status of the participant. Following the pain fMRI scan, two fMRI scans were performed for all the ALBP participants: one scan in the analgesic patch inducement and another scan in the algetic patch inducement pseudorandomly, with the continuous occurrence of ALBP through the scanning process.

To optimize the washout of the steady impacts triggered by the former intervention, the time interval between the two scans was 10 min. The pre- as well as the post-intervention changes in subjective VAS along with the fMRI signal changes induced by the identical post-intervention moderate pain stimuli function as the primary outcomes of this study.

**FIGURE 2:**
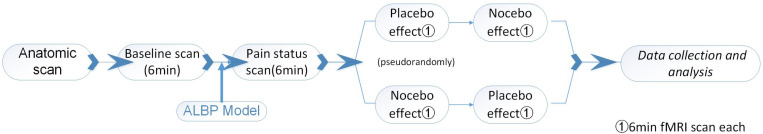
The experimental paradigm for the participants.

In the scanning, we instructed the participants to focus on the captions on the screen. When participants had an analgesic patch, the caption projected on the screen included “Please experience the effect of the analgesic patch, the scanning process is 6 min,” and when participants had an algetic patch, the caption projected on the screen included “Please experience the effect of the algetic patch, the scanning process is 6 min.” After the application of each stimulus, the VAS was projected on the screen, and the participants reported their pain scores (Figure 2).

### Imaging of the Brain

We carried out the experiment at the Department of Radiology of Zhujiang Hospital. A 3.0-T Philips Achieva MRI System (Royal Philips Electronics, Eindhoven, Netherlands) was employed to obtain the functional as well as the structural scans with an eight-channel head array coil designed for echo-planar imaging. The obtained images were axial as well as parallel to the bicommissural line, which covered the entire brain. Collection of the structural images was performed before the functional imaging was conducted using a T1-weighted fast spin echo sequence (matrix = 256 × 256, repetition time/echo time = 25/3 ms, thickness = 5 mm, slice = 24, slice gap = 0.7, flip angle = 30°, mm). Blood oxygenation level-dependent functional imaging was obtained *via* a T2^∗^-weighted, single-shot, gradient-recalled echo-planar imaging sequence (matrix = 64 × 64, repetition time/echo time = 2,000/35 ms, thickness = 5 mm, NSA = 1, slice = 24, slice gap = 0.7 mm, flip angle = 90°, 180 time points for a total of 360 s). Besides, fMRI image acquisition was preceded by five dummy scans to minimize gradient distortion.

### Definition of Seed Regions

**FIGURE 3:**
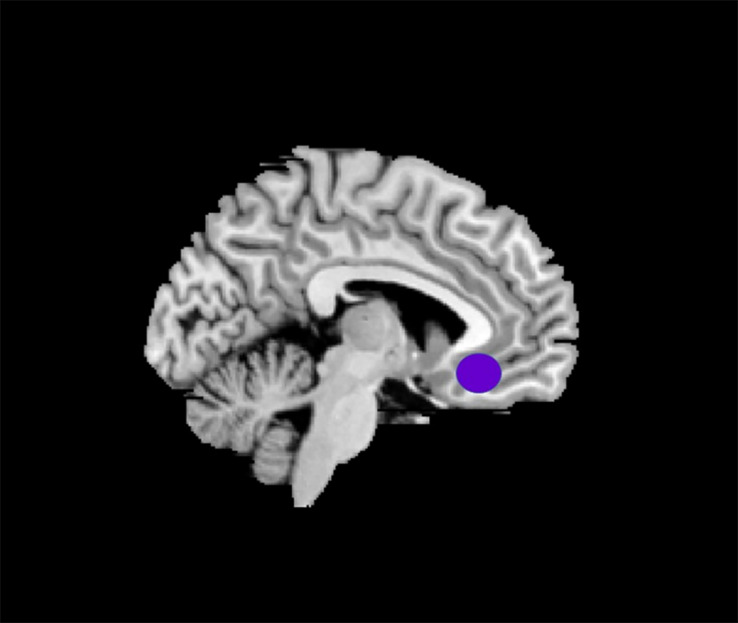
The location of ROI (rACC).

The data selection of the left side of rACC for the ROI (3 × 3 × 3 mm^3^) was based on the results of a previous MRI study (Margulies et al., 2007) for it to be on the same side with the intramuscular part. Montreal Neurological Institute (MNI) brain area coordinates were selected as the central voxel ROI (*x* = −5, *y* = 25, *z* = −10) (Figure 3).

### Resting-State Functional Connectivity Analysis

The Data Processing Assistant for Resting-State fMRI (DPARSF)^1^ was employed to pre-process and analyze the fMRI image data by the MATLAB R2013b routines. The pre-processing steps of the BOLD time series comprised removing the first 10 volumes, motion correction, normalization (3 × 3 × 3 mm^3^), slice-time correction to the Montreal Neurological Institute (MNI) templates, temporal band pass filtering, linear trend removal, and spatial smoothing. We adopted a function module (FC) of the REST software^2^.

This step was used to extract the individual time course of the activity from the areas relative to the standard EPI space for the rACC. We obtained brain functionality images for each participant through Pearson’s correlation coefficient analyses of the seed point as well as the whole brain voxel time series and standardization by Fisher’s *Z*-transformation of correlation coefficients into *z*-values.

### Behavioral Correlation Analysis

Based on the above statistical results, the brain areas of each key network in the female and male participant groups were included in the respective behavioral correlation analysis (BCA). Correlation analysis was performed between the pain score and each whole brain function connection signal map. The analysis was performed using the Correlation Analysis module of the REST software.

### Statistical Analyses

SPSS 18.0 software (SPSS, Chicago, IL, United States) was employed to analyze the descriptive statistics (mean ± SD) for the VAS and other data. The multivariate analysis of variance (MANOVA) and *t*-tests were used for statistical analysis. *p* < 0.05 signified statistical significance in accordance with an earlier stage of the trial.

The functional connectivity (FC) values between placebo response and pain status in the same group were computed *via* two-tailed, paired *t*-tests (*p* < 0.05), corrected for multiple comparisons [false discovery rate (FDR), *p* < 0.05].

Differences in the FC values of placebo response (or nocebo response) between the two groups were computed using two-tailed, two-sample *t*-tests (*p* < 0.05), corrected for multiple comparisons (FDR, *p* < 0.05).

In the BCA results, the correlation coefficient | *r*| > 0.60 and *p* < 0.05 were set as statistically significant.

## Results

In this study, 14 female participants (23.86 ± 2.41 years old) and 17 male participants (24.88 ± 2.98 years old) finally completed this research. The MANOVA results showed that the main effect (intervention factor) was statistically significant (*p* < 0.05; Table 1), and the interaction effect (intervention × sex group) was statistically significant (*p* < 0.05; Table 1). The follow-up *t*-test results showed that (1) in the group of male participants, the VAS scores were remarkably different between the placebo response and pain status (non-intervention status) and in the comparison of nocebo response and pain status (non-intervention status) (*p* < 0.05; Table 1); (2) similarly, in the group of female participants, there were VAS score differences between the placebo response and pain status (non-intervention status), as well as in the comparison of nocebo response and pain status (non-intervention status) (*p* < 0.05; Table 1); (3) besides, there were remarkable differences in VAS between male participants and female participants in placebo response, as well as in nocebo response (*p* < 0.05; Table 1); (4) nevertheless, there was no remarkable difference in the VAS scores of pain status between male participants and female participants (*p* > 0.05; Table 1).

**TABLE 1:** Summary of general data of the 31 participants.

VAS	Male (*n* = 17)	Female (*n* = 14)	*p*-value
Pain status	3.82 ± 0.64	3.86 ± 0.95	*p* = 0.907
Placebo response	2.29 ± 0.77^*a#*^	3.14 ± 0.86^a*^	*p* = 0.026
Nocebo response	4.65 ± 0.70^*b#*^	5.79 ± 1.19^b*^	*p* = 0.002

*F*_*intervention*_	96.605		*p* < 0.001
*F*_*intervention* × *sex group*_	4.777		*p* = 0.031

*^*a*^ Placebo response vs. pain status. ^*b*^ Nocebo response vs. pain status. *p*(a^#^) < 0.001, *p*(b^#^) = 0.038; *p*(a^∗^) = 0.003, *p*(b^∗^) < 0.001.*

### Resting-State FC Analysis

#### BOLD Signals of Placebo Response in Male Participants

**FIGURE 4:**
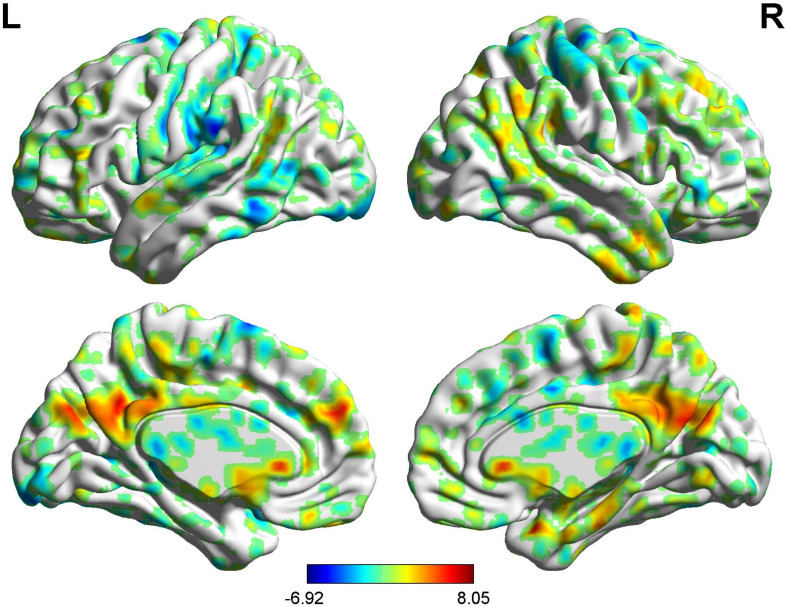
Brain areas showing functional connectivity with the rACC in placebo response in male participants. Color scale represents the *Z*-score. Red = placebo > pain; blue = pain > placebo.

**FIGURE 5:**
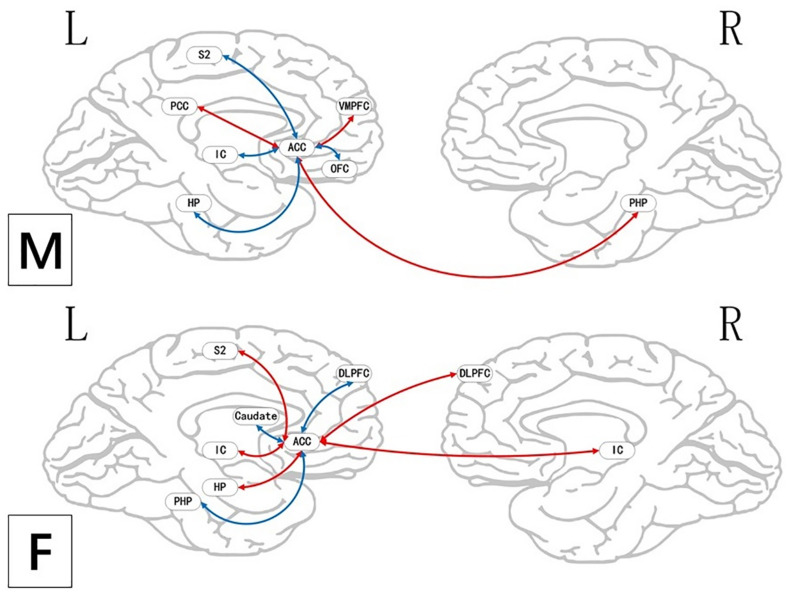
Brain areas indicating functional connectivity state differences in the two groups, when male or female participants (placebo response) compare their own pain status. M: Compared with female participants, male participants of placebo response had unique functional connectivity in placebo response; F: compared with male participants, female participants of placebo response had unique functional connectivity in placebo response. Some brain regions only showed projection position. Red = placebo > pain; blue = pain > placebo. L, left hemisphere; R, right hemisphere; OFC, orbitofrontal cortex; DLPFC, dorsolateral prefrontal cortex; VMPFC, ventromedial prefrontal cortex; TP, temporal pole; HP, hippocampus gyrus; PHP, parahippocampal gyrus; pgACC, pregenual anterior cingulate cortex; IC, insular; MCC, mid-cingulate cortex; PCC, posterior cingulate cortex; THS, thalamus; SMA, supplementary motor area; S1, primary somatosensory area; S2, secondary somatosensory area; AG, angular gyrus.

In the FC map of placebo response, we find that rACC displayed increased FC in the posterior cingulate cortex (PCC), parahippocampal gyrus (PHP), brainstem, angular gyrus (AG), ventromedial prefrontal cortex (VMPFC), and primary somatosensory area (S1). Besides, the rACC exhibited remarkably decreased FC with the pregenual anterior cingulate cortex (pgACC), cerebellum, orbitofrontal cortex (OFC), hippocampal gyrus (HP), IC, secondary somatosensory area (S2), and supplementary motor area (SMA) (Table 2 and Figures 4, 5).

**TABLE 2:** Summary of the brain areas indicating functional connectivity with the rACC in placebo response in male participants (*p* < 0.05, FDR < 0.05).

Brain region	R/L	MNI			Voxel	*Z*-score
		*x*	*y*	*z*		
Cerebellum posterior lobe	L	−42	−57	−33	121	−6.9151
Brainstem	R	6	−18	−15	51	5.5208
VMPFC	L	−3	54	30	56	6.8665
OFC	L	−12	24	−27	66	−4.7077
pgACC	R	21	24	27	60	−5.2751
pgACC	L	−21	36	12	97	−5.3435
PCC	L	−3	−72	24	712	7.1829
PHP	R	21	−24	−36	127	6.0958
HP	L	−42	−54	3	215	−6.5079
IC	L	−24	−15	21	118	−5.8694
S1	L	−36	−9	36	134	5.3472
S2	L	−51	−15	18	191	−6.8008
SMA	R	6	6	57	52	−5.3626
AG	R	57	−66	15	126	5.6137

*Abbreviations: FDR, false discovery rate; MNI, Montreal Neurological Institute; OFC, orbitofrontal cortex; DLPFC, dorsolateral prefrontal cortex; VMPFC, ventromedial prefrontal cortex; TP, temporal pole; HP, hippocampus gyrus; PHP, parahippocampal gyrus; pgACC, pregenual anterior cingulate cortex; IC, insular; MCC, mid-cingulate cortex; PCC, posterior cingulate cortex; THS, thalamus; SMA, supplementary motor area; S1, primary somatosensory area; S2, secondary somatosensory area; AG, angular gyrus.*

### BOLD Signals of Placebo Response in Female Participants

**FIGURE 6:**
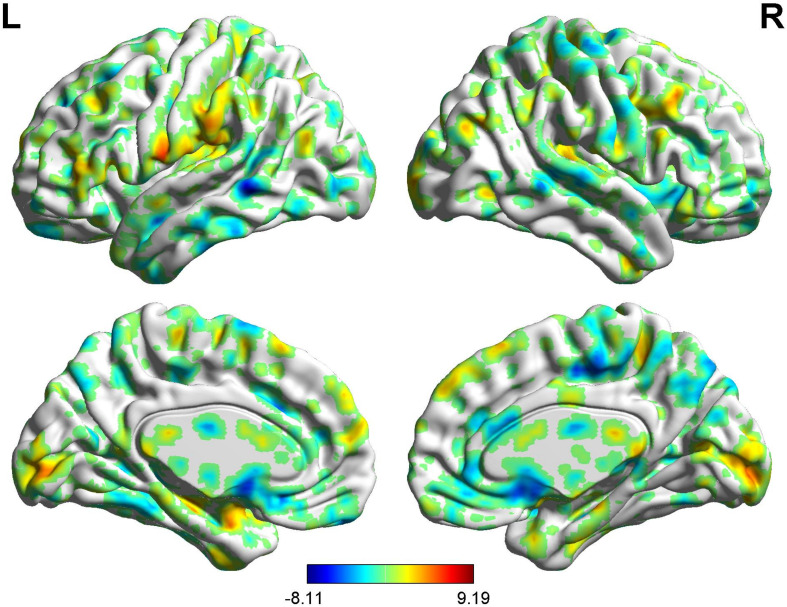
Brain areas indicating functional connectivity with the rACC in placebo response in female participants. Color scale represents the *Z*-score. Red = placebo > pain; blue = pain > placebo.

In the FC map of placebo response, we find that rACC displayed increased FC in the lingual gyrus, brainstem, r-DLPFC, HP, IC, and S2. Besides, the rACC exhibited remarkably decreased FC with the cerebellum, l-DLPFC, pgACC, caudate, PHP, and SMA (Table 3 and Figures 6, 5).

**TABLE 3:** Summary of the brain areas indicating functional connectivity with the rACC in placebo response in female participants (*p* < 0.05, FDR < 0.05).

Brain region	R/L	MNI			Voxel	*Z*-score
		*x*	*y*	*z*		
Cerebellum posterior lobe	R	39	−69	−24	110	−5.8215
Brainstem	L	−3	−27	−21	53	5.7238
Lingual gyrus	R	15	−90	0	233	6.2514
DLPFC	R	36	12	36	67	5.4365
DLPFC	L	−33	51	0	83	−5.1417
pgACC	L	−15	21	27	95	−6.7006
pgACC	R	18	33	27	68	−4.6017
Caudate	L	−6	9	−9	274	−6.6087
PHP	L	−30	−45	−3	67	−5.4339
HP	L	−36	0	−27	74	6.2651
IC	R	45	−15	0	165	9.1888
IC	L	−48	−15	9	71	7.1934
S2	L	−66	−15	12	135	6.7534
SMA	R	9	−12	48	61	−6.999

*Abbreviations: FDR, false discovery rate; MNI, Montreal Neurological Institute; OFC, orbitofrontal cortex; DLPFC, dorsolateral prefrontal cortex; VMPFC, ventromedial prefrontal cortex; TP, temporal pole; HP, hippocampus gyrus; PHP, parahippocampal gyrus; pgACC, pregenual anterior cingulate cortex; IC, insular; MCC, mid-cingulate cortex; PCC, posterior cingulate cortex; THS, thalamus; SMA, supplementary motor area; S1, primary somatosensory area; S2, secondary somatosensory area; AG, angular gyrus.*

#### BOLD Signals of Placebo Response (Males vs. Females)

**FIGURE 7:**
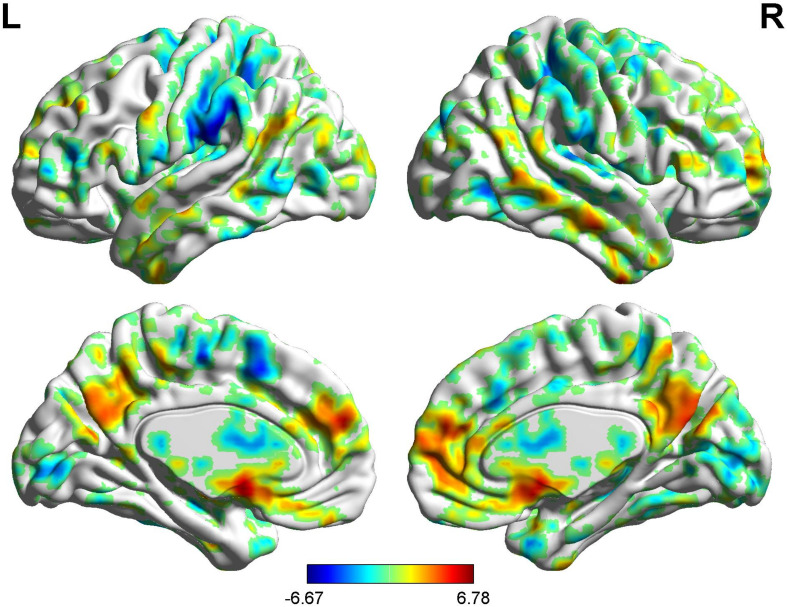
Brain areas showing functional connectivity with the rACC in comparison of male participants and female participants in placebo response. Color scale represents the *Z*-score. Red = male > female; blue = female > male.

In the FC map of placebo response, we find that in the placebo response, the male group displayed increased FC in the cerebellum, brainstem, VMPFC, PCC, caudate, TP, superior temporal lobe, and AG compared with the female group. Additionally, the rACC exhibited remarkably decreased FC with the THS, IC, S1, S2, and SMA (Table 4 and Figure 7).

**TABLE 4:** Summary of the brain areas showing functional connectivity with the rACC in comparison of male participants and female participants in placebo response (*p* < 0.05, FDR < 0.05).

Brain region	R/L	MNI			Voxel	*Z*-score
		*x*	*y*	*z*		
Cerebellum posterior lobe	R	51	−57	−48	75	4.5541
Brainstem	L	−3	−39	−6	66	5.6247
VMPFC	L	−3	54	24	499	6.7631
PCC	R	3	−75	30	421	4.9952
Caudate	L	−3	6	−9	197	6.7788
THS	R	21	−27	0	68	−5.1253
TP	R	45	−3	−48	64	5.4192
Superior temporal lobe	R	66	−54	18	118	4.9247
IC	R	45	−18	0	261	−5.6628
S1	R	30	−36	63	186	−6.0444
S1	L	−30	−30	57	113	−5.3669
S2	L	−63	−30	27	498	−6.6698
SMA	L	−6	−15	54	114	−5.0419
AG	L	−51	−63	30	61	3.781

*Abbreviations: FDR, false discovery rate; MNI, Montreal Neurological Institute; OFC, orbitofrontal cortex; DLPFC, dorsolateral prefrontal cortex; VMPFC, ventromedial prefrontal cortex; TP, temporal pole; HP, hippocampus gyrus; PHP, parahippocampal gyrus; pgACC, pregenual anterior cingulate cortex; IC, insular; MCC, mid-cingulate cortex; PCC, posterior cingulate cortex; THS, thalamus; SMA, supplementary motor area; S1, primary somatosensory area; S2, secondary somatosensory area; AG, angular gyrus.*

#### BOLD Signals of Nocebo Response in Male Participants

**FIGURE 8:**
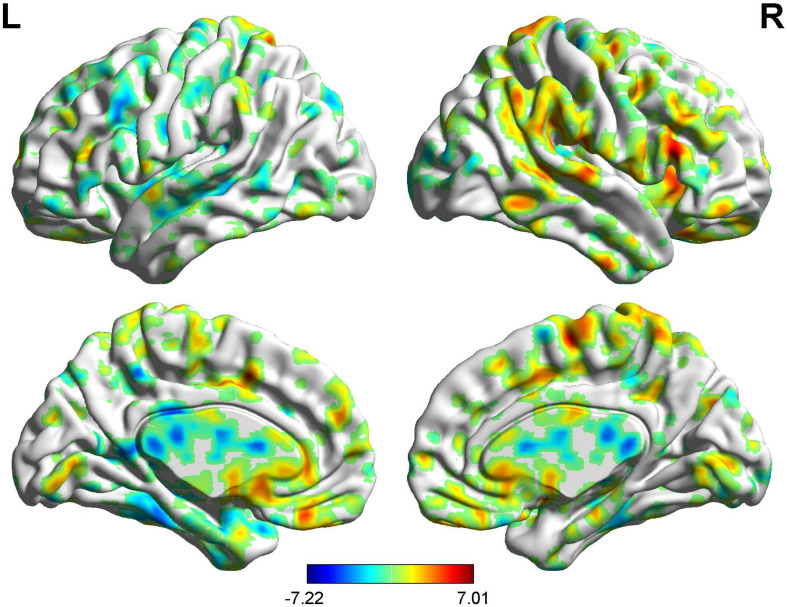
Brain areas showing functional connectivity with the rACC in nocebo response in male participants. Color scale represents the *Z*-score. Red = nocebo > pain; blue = pain > nocebo.

**FIGURE 9:**
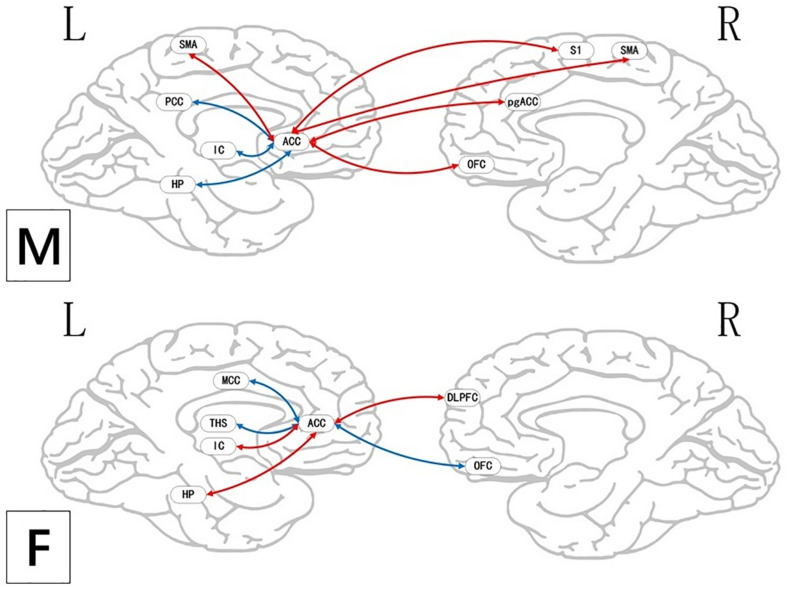
Regions showing significantly functional connectivity state differences in the two groups, when male or female participants (nocebo response) compare their own pain status. M: Compared with female participants, male participants of nocebo response had unique functional connectivity in the brain network; F: compared with male participants, female participants of nocebo response had unique functional connectivity in the brain network. Some brain regions only showed projection position. Red = nocebo > pain; blue = pain > nocebo. L, left hemisphere; R, right hemisphere; OFC, orbitofrontal cortex; DLPFC, dorsolateral prefrontal cortex; VMPFC, ventromedial prefrontal cortex; TP, temporal pole; HP, hippocampus gyrus; PHP, parahippocampal gyrus; pgACC, pregenual anterior cingulate cortex; IC, insular; MCC, mid-cingulate cortex; PCC, posterior cingulate cortex; THS, thalamus; SMA, supplementary motor area; S1, primary somatosensory area; S2, secondary somatosensory area; AG, angular gyrus.

In the FC map of nocebo response, we find that rACC displayed increased FC in the OFC, pgACC, r-IC, S1, S2, and SMA. Besides, the rACC had remarkably decreased FC with the cerebellum, PCC, PHP, HP, and l-IC (Table 5 and Figures 8, 9).

**TABLE 5:** Summary of the brain regions indicating functional connectivity with the rACC in nocebo response in male participants (*p* < 0.05, FDR < 0.05).

Brain region	R/L	MNI			Voxel	*Z*-score
		*x*	*y*	*z*		
Cerebellum anterior lobe	L	−6	−51	−24	447	−6.6399
OFC	R	15	24	−18	266	5.2666
pgACC	R	27	36	6	124	6.4451
PCC	L	−33	−15	24	663	−6.4104
PHP	L	−39	−42	−18	161	−6.0527
HP	L	−36	−36	−6	151	−4.8558
IC	R	30	18	3	397	7.0053
IC	L	−33	−3	−9	193	−5.4464
S1	R	18	−45	75	80	5.0374
S2	R	66	−18	6	434	5.884
SMA	L	−9	9	45	103	5.6286
SMA	R	9	−9	63	161	5.9196

*Abbreviations: FDR, false discovery rate; MNI, Montreal Neurological Institute; OFC, orbitofrontal cortex; DLPFC, dorsolateral prefrontal cortex; VMPFC, ventromedial prefrontal cortex; TP, temporal pole; HP, hippocampus gyrus; PHP, parahippocampal gyrus; pgACC, pregenual anterior cingulate cortex; IC, insular; MCC, mid-cingulate cortex; PCC, posterior cingulate cortex; THS, thalamus; SMA, supplementary motor area; S1, primary somatosensory area; S2, secondary somatosensory area; AG, angular gyrus.*

#### BOLD Signals of Nocebo Response in Female Participants

**FIGURE 10:**
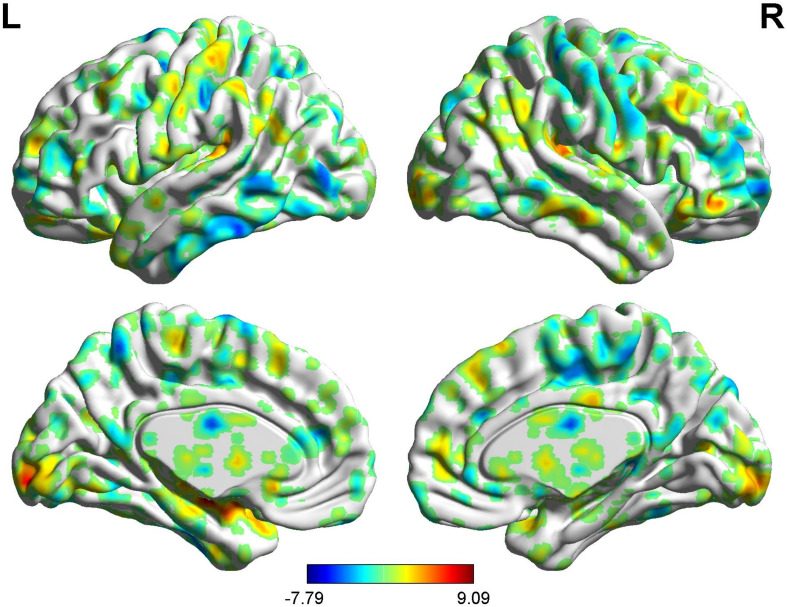
Brain areas showing functional connectivity with the rACC in nocebo response in female participants. Color scale represents the *Z*-score. Red = nocebo > pain; blue = pain > nocebo.

In the FC map of nocebo response, we find that rACC displayed increased FC in the cerebellum, brainstem, lingual gyrus, DLPFC, HP, IC, and S2. In addition, the rACC exhibited significantly decreased FC with the OFC, MCC, THS, and PHP (Table 6 and Figures 10, 9).

**TABLE 6:** Summary of the brain regions indicating functional connectivity with the rACC in nocebo response in female participants (*p* < 0.05, FDR < 0.05).

Brain region	R/L	MNI			Voxel	*Z*-score
		*x*	*y*	*z*		
Cerebellum anterior lobe	R	15	−36	−27	82	5.5554
Brainstem/pons	R	3	−24	−24	119	5.6579
Lingual gyrus	L	−12	−96	−6	335	9.0865
DLPFC	R	45	45	−9	57	6.9047
OFC	R	24	66	−3	82	−5.5828
MCC	L	−24	−24	45	507	−6.171
THS	L	−3	−12	21	63	−6.202
PHP	L	−42	−18	−33	358	−7.7163
HP	L	−27	−12	−15	312	8.0313
IC	L	−27	−42	18	199	8.7099
IC	R	42	−39	18	141	5.7512
S2	R	45	21	39	86	5.2254

*Abbreviations: FDR, false discovery rate; MNI, Montreal Neurological Institute; OFC, orbitofrontal cortex; DLPFC, dorsolateral prefrontal cortex; VMPFC, ventromedial prefrontal cortex; TP, temporal pole; HP, hippocampus gyrus; PHP, parahippocampal gyrus; pgACC, pregenual anterior cingulate cortex; IC, insular; MCC, mid-cingulate cortex; PCC, posterior cingulate cortex; THS, thalamus; SMA, supplementary motor area; S1, primary somatosensory area; S2, secondary somatosensory area; AG, angular gyrus.*

#### BOLD Signals of Nocebo Response (Males vs. Females)

**FIGURE 11:**
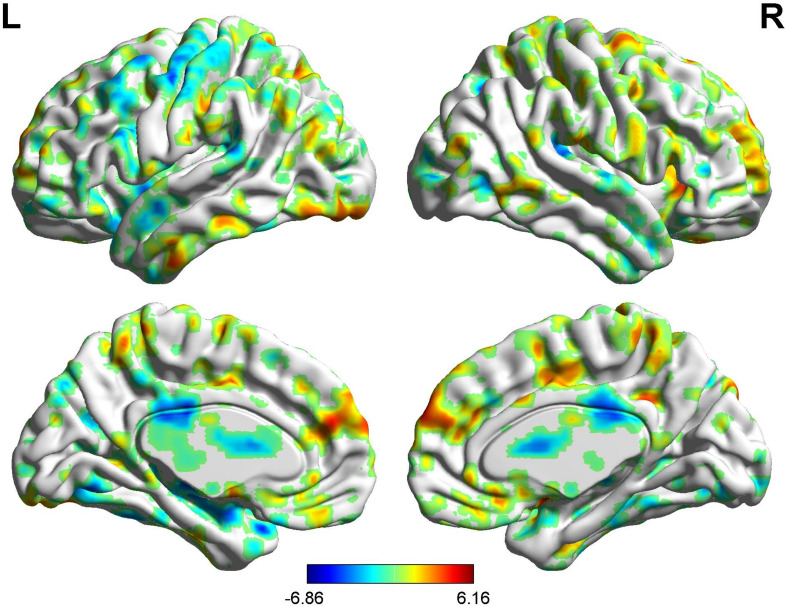
Brain areas showing functional connectivity with the rACC in comparison of male participants and female participants in nocebo response. Color scale represents the *Z*-score. Red = male > female; blue = female > male.

In the FC map of the rACC, the findings show that for nocebo response, the male group displayed increased FC in the OFC, VMPFC, DLPFC, S1, S2, and SMA compared with the female group. Additionally, the rACC exhibited remarkably decreased FC with the cerebellum, PCC, TP, putamen, and IC (Table 7 and Figure 11).

**TABLE 7:** Summary of the brain areas showing functional connectivity with the rACC in comparison of male participants and female participants in nocebo response (*p* < 0.05, FDR < 0.05).

Brain region	R/L	MNI			Voxel	*Z*-score
		*x*	*y*	*z*		
Cerebellum anterior lobe	R	18	−48	−24	592	−6.5656
OFC	R	9	24	−21	52	4.2662
VMPFC	R	3	51	24	226	5.5423
DLPFC	R	36	36	−12	126	5.359
PCC	L	−27	−42	18	418	−6.8637
TP	L	−36	15	−27	94	−6.3299
TP	R	45	0	−18	94	−6.1386
Putamen	R	24	−12	0	85	−5.0311
IC	L	−33	0	−9	268	−6.5591
S1	R	24	−3	72	60	4.3852
S1	L	−36	−6	42	153	4.8499
S2	L	−51	−27	30	82	5.2059
SMA	R	9	0	39	62	3.5299

*Abbreviations: FDR, false discovery rate; MNI, Montreal Neurological Institute; OFC, orbitofrontal cortex; DLPFC, dorsolateral prefrontal cortex; VMPFC, ventromedial prefrontal cortex; TP, temporal pole; HP, hippocampus gyrus; PHP, parahippocampal gyrus; pgACC, pregenual anterior cingulate cortex; IC, insular; MCC, mid-cingulate cortex; PCC, posterior cingulate cortex; THS, thalamus; SMA, supplementary motor area; S1, primary somatosensory area; S2, secondary somatosensory area; AG, angular gyrus.*

### Brain Response of BCA of Placebo Response

**FIGURE 12:**
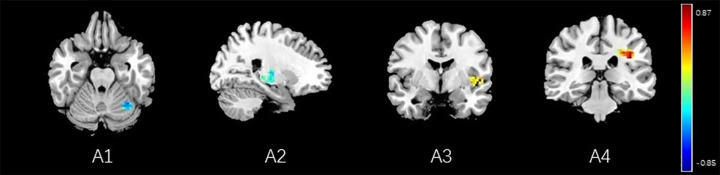
Regions showing significant correlation with the pain score of placebo response in female participants. Color scale represents the correlation coefficient. Red = positive correlation; blue = negative correlation. A1: cerebellum posterior lobe, A2: thalamus, A3: insular, A4: secondary somatosensory area.

**FIGURE 13:**
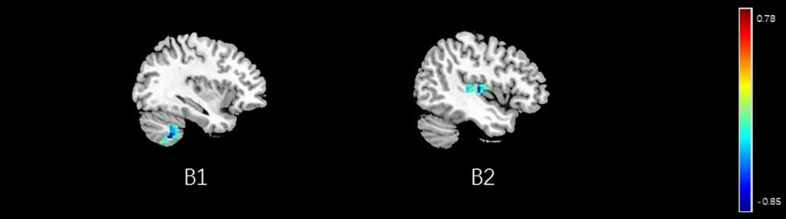
Regions showing significant correlation with the pain score of placebo response in male participants. Color scale represents the correlation coefficient. Red = positive correlation; blue = negative correlation. B1: cerebellum posterior lobe, B2: insular.

Correlation analysis between pain scores and FC maps showed that placebo analgesia was positively correlated to FC between the rACC and the IC as well as between the rACC and the S2 and placebo analgesia was negatively correlated to FC between the rACC and the cerebellum as well as between the rACC and the THS in female participants. On the other hand, placebo analgesia was negatively correlated to FC between the rACC and the cerebellum as well as between the rACC and IC in male participants (see Table 8 and Figures 12, 13).

**TABLE 8:** Analysis of the correlation between pain score and functional connectivity in placebo response (| *r*| > 0.60, *p* < 0.05).

Brain region	R/L	MNI			Voxel	*r*
		*x*	*y*	*z*		
**Female**						
Cerebellum posterior lobe	L	−33	−60	−18	63	−0.77
THS	R	27	−21	−6	71	−0.85
IC	L	−51	−3	0	127	0.85
S2	L	−39	−30	27	233	0.87
**Male**						
Cerebellum posterior lobe	L	−36	−51	−51	77	−0.83
IC	L	−45	−18	6	115	−0.81

*Abbreviations: MNI, Montreal Neurological Institute; IC, insular; THS, thalamus; S2, secondary somatosensory area.*

### Brain Response of BCA of Nocebo Response

**FIGURE 14:**
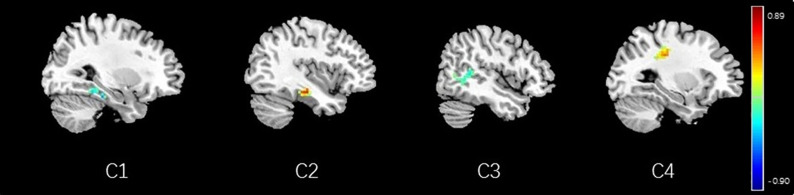
Regions showing significant correlation with the pain score of nocebo response in female participants. Color scale represents the correlation coefficient. Red = positive correlation; blue = negative correlation. C1: right parahippocampal gyrus, C2: left parahippocampal gyrus, C3: middle temporal lobe, C4: secondary somatosensory area.

**FIGURE 15:**
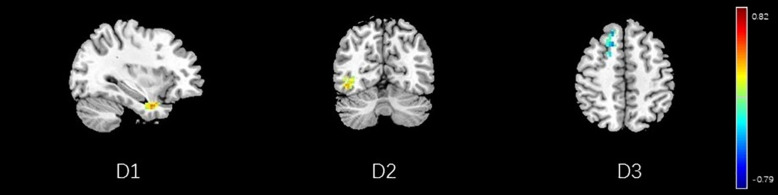
Regions showing significant correlation with the pain score of nocebo response in male participants. Color scale represents the correlation coefficient. Red = positive correlation; blue = negative correlation. D1: temporal pole, D2: inferior temporal lobe, D3: dorsolateral prefrontal cortex.

Correlation analysis between pain scores and FC maps showed that nocebo hyperalgesia was positively correlated to FC between the rACC and the left PHP as well as between the rACC and the S2 and placebo analgesia was negatively correlated to FC between the rACC and the right PHP as well as between the rACC and the middle temporal lobe in female participants. On the other hand, nocebo hyperalgesia was positively correlated to FC between the rACC and the TP as well as between the rACC and the inferior temporal lobe, and nocebo hyperalgesia was negatively correlated to FC between the rACC and the DLPFC in male participants (see Table 9 and Figures 14, 15).

**TABLE 9:** Analysis of the correlation between pain score and functional connectivity in nocebo response (| *r*| > 0.60, *p* < 0.05).

Brain region	R/L	MNI			Voxel	*r*
		*x*	*y*	*z*		
**Female**						
PHP	R	36	−42	−21	65	−0.79
PHP	L	−39	−21	−18	132	0.79
Middle temporal lobe	R	57	−63	9	131	−0.83
S2	L	−30	−30	33	66	0.75
**Male**						
TP	L	−36	−6	−30	53	0.71
Inferior temporal lobe	R	45	−75	−6	77	0.74
DLPFC	R	15	21	54	81	−0.71

*Abbreviations: MNI, Montreal Neurological Institute; DLPFC, dorsolateral prefrontal cortex; PHP, parahippocampal gyrus; S2, secondary somatosensory area; TP, temporal pole.*

## Discussion

Here, we explored the impact of sex on nocebo response in the ALBP model and nocebo responses in the same group for the first time.

In the present study, we found VAS score differences between male and female participants. Moreover, there were BOLD signal differences between the two groups, demonstrating that brain network features differed between the two study groups. The BCA results also showed differences in the correlation coefficients of several brain areas between the two study groups.

### Sex Differences in Placebo Response

During the placebo condition, we found FC differences in the brain networks of the two groups. In male participants, the rACC had greatly reduced FC with the OFC, pgACC, HP, IC, S2, and SMA, while in female participants, reduced FC was apparent only with the DLPFC, pgACC, and SMA. The activated regions in the pain status are called the pain-associated network (Apkarian et al., 2005; Tracey, 2008), which mainly includes the ACC, PFC, S1, IC, S2, and THS (Tracey, 2008). Our results suggest that under the effect of placebo, the pain-associated network is involved in the acquisition and processing of sensory signals and reduces the excitability of related brain regions (Ingvar, 1999). In the male participants, the activity of the pain-associated network was lower relative to that in the female participants, suggesting the sex effects found in the placebo response.

It is worth noting that, as the key node of sensory transmission, IC transmits sensory signals from THS and then transmits them to the anterior cingulate gyrus to complete sensory information processing (Craig et al., 2000). The IC is also of major significance for interoception as well as multimodal sensory integration regarding pain and has a vital role in pain-associated decision-making (Katja et al., 2010) and emotional awareness, along with the integration of (anticipated) interoceptive stimuli (Craig, 2009). Our results showed that there was lower FC value between IC and rACC in the male group of the placebo analgesia. Similarly, we also found a negative correlation between IC and VAS score in BCA results of the male group. It suggests that placebo effect may affect the capacity to cognitive processing at the cortical level, so as to decrease pain-linked emotional and “dampen” painful afferent sensory information. Female participants, in contrast, may have less capacity of this cognitively driven decreased FC of IC in the placebo analgesia.

At the same time, we should also note that, during the placebo condition, the rACC showed increased FC with the brainstem, VMPFC, PCC, and PHP in male participants. These brain areas are closely related to the activity of the endogenous opioid system (Vambheim and Flaten, 2017) and could activate it. Thus, the stronger placebo responses in males may be the result of a more effective endogenous opioid system in males compared with that in females. The reward system constitutes a group of neural structures, which are responsible for the associative learning, incentive salience, and positive emotions, especially the ones that entail pleasure as a central component (Berridge and Kringelbach, 2015; Schultz, 2015). It includes the ventral striatum, PFC, ACC, IC, HP, THS, and dorsal striatum, among other areas (Berridge and Kringelbach, 2015). Our findings demonstrated that the rACC had elevated FC with DLPFC, HP, and IC in female participants, demonstrating that the reward system may be remarkably activated in placebo analgesia in females. Moreover, we also found a negative correlation between THS and VAS score in female participants. It also suggests that the reward system may be intimately involved in placebo analgesia and that the analgesic effect may be influenced by the intensity of activation of this system. The activation of the reward system produces higher amounts of neurotransmitters, such as dopamine and GABA (Yager et al., 2015), which can promote happy moods and reduce feelings of pain. At the same time, compared with female participants, the male participants showed widely reduced FC with the pain matrix.

### Sex Differences in Nocebo Response

When a situation is presented, HP shows a strong association with the approach–avoidance conflict which is either punishing or rewarding, implying that the subsequent decision-making is linked to anxiety (O’Neil et al., 2015). Anxiety is regarded as a central cause of the nocebo response (Thibodeau et al., 2013). During the nocebo condition, our results showed that the rACC had decreased FC with HP in male participants and increased FC with HP in female participants. The difference of FC with HP in different groups may be related to the level of anxiety, which may play a role in the nocebo response. The above phenomenon reflects the sex difference of nocebo response, and the relationship between HP and anxiety needs to be further explored. In female participants, the left PHP was positively correlated with VAS score, while the right PHP was negatively correlated with VAS score. The PHP is known to be involved in memory encoding and retrieval (Rosner et al., 2013), as well as in the construction of networks in the limbic system (Rajmohan and Mohandas, 2007). Our results suggest a characteristic of the nocebo response in female subjects, and the reasons for this difference need to be further explored.

During the nocebo condition, we established that the pain matrix had remarkably elevated FC in both male and female participants. This is in agreement with heightened pain sensation. In contrast with placebo responses, under the nocebo response, the brain escalates the transmission along with the analysis of pain information, causing it to generate more pain sensations. The increased FC between the rACC and IC indicates that the nocebo response may improve the sensory information transfer function of the IC and may simultaneously increase the sensory information processing speed of the ACC, hence resulting in the hyperalgesia effect.

Research has revealed a strong relationship of the nocebo response with the anxiety triggered by the cholecystokinin (CCK) (Skibicka and Dickson, 2013; Carlino et al., 2017). Behavioral studies have documented the dominant role of CCK in nocebo hyperalgesia *via* anticipatory anxiety mechanisms (Zwanzger et al., 2012). Other studies have documented that CCK release is strongly linked to the ACC, HP, AMYG, IC, THS, and other brain areas (Zwanzger et al., 2012). Herein, we demonstrated that the rACC had increased FC with the HP and IC, replicating the role of anxiety in the nocebo response. The connectivity of the OFC, as the pivotal nerve center of emotion regulation, is completely opposite in the two sex groups: in male participants, the increased FC between the rACC and OFC may affect the function of emotion control and reduce anxiety; conversely, females may experience the nocebo response more intensely because of the possible uncontrolled anxiety. Moreover, compared with female participants, in male participants, the rACC showed wide increased FC with the PFC, including the VMPFC, OFC, and DLPFC. These results suggest that males may speed up the processing of sensory information and may have better control over their emotions, thereby reducing the effects of the nocebo. In the BCA results, the negative correlation between DLPFC and VAS score also suggested that the above accelerated processes might reduce the effect of nocebo response and play a role in sex differences of nocebo hyperalgesia.

### Limitation

Although the results discussed herein demonstrate the sex differences between nocebo hyperalgesia and placebo analgesia, the study has three limitations. Firstly, the use of rs-fMRI alone was too monotonous. An integration of task-fMRI the event-linked fMRI could yield findings that are more abundant. Secondly, only young people were enrolled in the study; therefore, the differences between participants of different ages were not explored. Thirdly, due to the limitation of study design and the small sample size of female participants in the study, it was difficult to include the factors of menstrual cycle into the analysis, and the effects of menstrual cycle-related hormones (such as estradiol and progesterone) are still worthy of attention.

## Conclusion

In this study, we found that the endogenous opioid system and reward circuit have a vital role in sex differences of placebo response and that anxiety and its secondary reactions may cause the sex differences of nocebo response.

## Data Availability Statement

The raw data supporting the conclusions of this article will be made available by the authors, without undue reservation.

## Ethics Statement

The studies involving human participants were reviewed and approved by Ethics Committee of Zhujiang Hospital. The patients/participants provided their written informed consent to participate in this study.

## Author Contributions

YS, HZ, and WW: study concept and design. YS, YZ, and SH: acquisition, analysis, or interpretation of data. YS and WW: drafting of the manuscript. JY and WW: critical revision of the manuscript and study supervision. YS, YZ, GC, and SH: statistical analysis. JY: MRI technical support. All authors contributed to the article and approved the submitted version.

## Conflict of Interest

The authors declare that the research was conducted in the absence of any commercial or financial relationships that could be construed as a potential conflict of interest.

## Publisher’s Note

All claims expressed in this article are solely those of the authors and do not necessarily represent those of their affiliated organizations, or those of the publisher, the editors and the reviewers. Any product that may be evaluated in this article, or claim that may be made by its manufacturer, is not guaranteed or endorsed by the publisher.

## Footnotes

1
http://www.restfmri.net

2
http://restfmri.net/forum/rest
